# Angioleiomyoma of the pulmonary artery: a case report and literature review

**DOI:** 10.1186/s13019-020-01275-z

**Published:** 2020-08-28

**Authors:** Yan Hu, Siying Ren, Sichuang Tan, Chen Chen, Xiang Wang, Qingchun Liang, Fenglei Yu, Wenliang Liu

**Affiliations:** 1 Department of Thoracic Surgery, Second Xiangya Hospital of Central South University, No.139 Renmin Road, Changsha, 410011 China; 2 Department of Respiratory and Critical Care Medicine, Second Xiangya Hospital of Central South University, Changsha, 410011 China; 3 Department of Pathology, Second Xiangya Hospital of Central South University, Changsha, 410011 China

**Keywords:** Angioleiomyoma, Lung neoplasm, Differential diagnosis, Case report

## Abstract

**Background:**

Angioleiomyoma of the pulmonary artery is rare in the literature and few studies have been reported. Here we present a rare case of angioleiomyoma arising from the pulmonary artery in a young patient.

**Case presentation:**

A 27-year-old male patient presented to our clinic due to the incidental finding of a nodule in the right lower lobe of the lung, which was unchanged from the prior year. Preoperative CT scans showed a well-demarcated nodule of soft tissue density penetrated by the basal branch of the right anterior basilar artery (RA8b). Single-port video-assisted RS8 segmentectomy was performed under the guidance of preoperative 3-dimensional reconstruction for histologic confirmation of the tumour. The tumour appeared as a solid tumour of a tube-like structure with vascular endothelium, composed of spindle-shaped smooth muscle cells lacking nuclear atypia and homogenous red-dye substances. The spindle cells were positive for immunostaining for smooth muscle actin (SMA), desmin and Ki-67 and were negative for immunostaining for Dog-1, HMB45, and Melan-A. A pathological diagnosis of primary angioleiomyoma of the pulmonary artery was finally made.

**Conclusions:**

This report is a reminder for thoracic surgeons that angioleiomyoma should be included in the differential diagnosis of lung neoplasms, especially for the mass of soft tissue density penetrated by pulmonary blood vessels shown by CT. Awareness of this rare entity should potentially prevent underdiagnosis and improper surgical treatment.

## Background

Angioleiomyoma is a benign soft tissue tumour comprising mature smooth muscle cells with a prominent vascular component [1]. It typically forms a well-circumscribed, subcutaneous nodule less than 20 mm in adults, especially in the leg [2]. However, this type of tumour rarely occurs in the lung. We herein report an interesting case of angioleiomyoma arising from the pulmonary artery and present a literature review of previously reported cases.

## Case presentation

**Fig. 1:**
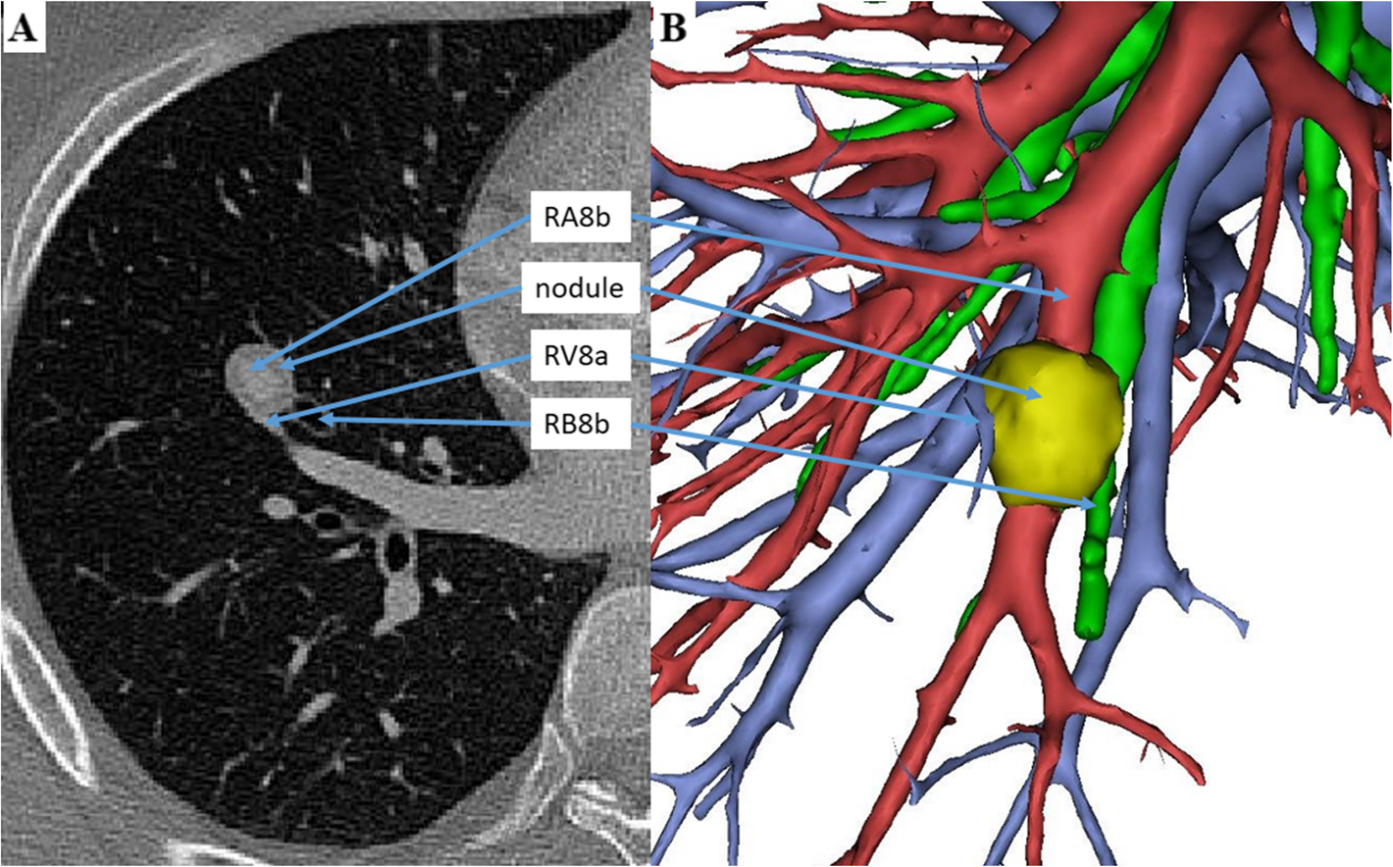
Findings of CT and 3D reconstruction in a patient with angioleiomyoma of the pulmonary artery. Contrast-enhanced computed tomography (CT) of the chest showed a well-demarcated mass with a maximum diameter of 1.7 cm in the right anterior basilar segment of the lung (RS8), adjacent to the basal branch of the right anterior basilar bronchus (RB8b). The lesion, displaying soft tissue density in the periphery, was penetrated by the basal branch of the right anterior basilar artery (RA8b) and bypassed by the lateral branch of the right anterior basilar vein (RV8a). Preoperative 3-dimensional (3D) reconstruction of the pulmonary vessels and bronchial trees of the right lower lobe from CT images was performed, and single-port RS8 segmentectomy was performed for histologic confirmation of the mass

A 27-year-old male patient complained of the incidental finding of a nodule in the right lower lobe of the lung on a routine health examination in October 2019, which was unchanged on repeat examinations from the prior year. No related subjective symptoms were noted and the physical examination showed no abnormalities. The results of routine blood biochemistry, oncological biomarkers and flexible bronchoscopy were normal. Contrast-enhanced computerized tomography of the chest showed a well-demarcated mass with a maximum diameter of 1.7 cm in the right anterior basilar segment of the lung (RS8). The lesion, which displayed a soft tissue density in the periphery, was penetrated by the basal branch of the right anterior basilar artery (RA8b) (Fig. 1).

**Fig. 2:**
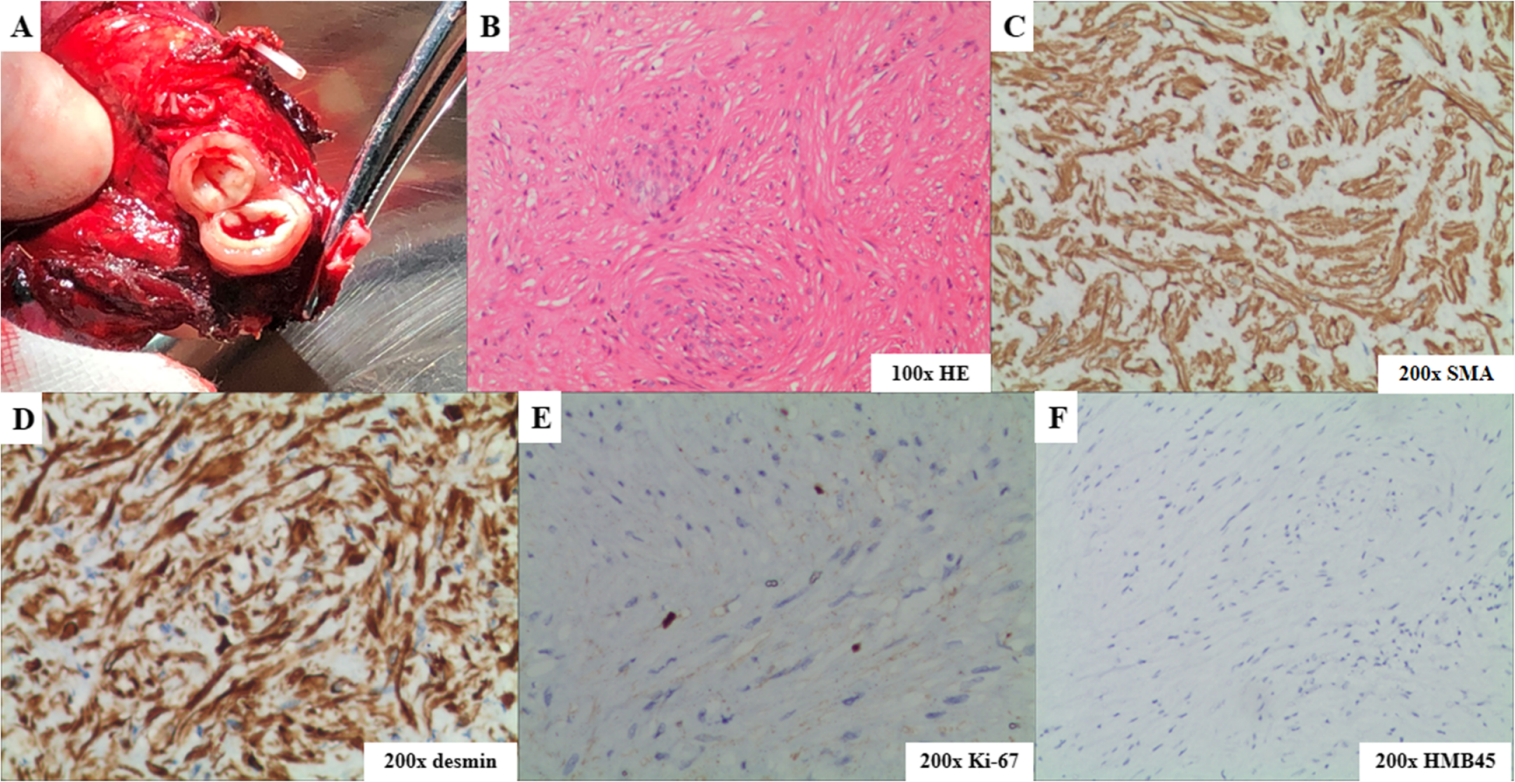
Gross appearance and microscopic pathology of the tumour. **a**, After dividing the mass, it appeared as a solid tumour exhibiting a tube-like structure with vascular endothelium; **b**, Histologically, the mass was displayed as proliferating spindle-shaped smooth muscle cells lacking nuclear atypia and homogenous red-dye substances; **c**, the spindle cells showed positive immunostaining for smooth muscle actin (SMA); **d**, the spindle cells showed positive immunostaining for desmin; **e**, the spindle cells showed positive immunostaining for Ki-67 (labelling 3%); **f**, the spindle cells showed negative immunostaining for HMB45

Preoperative 3-dimensional reconstruction of the pulmonary vessels and bronchial trees of the right lower lobe from CT images was performed for surgical guidance (Fig. 1) and surgical resection was performed for histologic confirmation of the tumour. In view of its centrally-located site and suspicion of a benign lesion, single-port video-assisted RS8 segmentectomy was performed. The well-bounded nodule was resected with clear margins. After dividing the lesion, it appeared as a solid tumour of a tube-like structure with vascular endothelium (Fig. 2), composed of spindle-shaped smooth muscle cells lacking nuclear atypia and homogenous red-dye substances. Focal calcification was noted and no malignant evidence was detected. The spindle cells exhibited positive immunostaining for smooth muscle actin (SMA), desmin and Ki-67 (labelling 3%) and negative immunostaining for Dog-1, HMB45, and Melan-A (Fig. 2). The patient experienced an uneventful postoperative course. Immediate postoperative radiographs on postoperative day 3 showed complete reexpansion of the lung without obvious pneumothorax or hydrothorax. At his 6-month follow-up, the patient was well without complications or evidence of recurrence.

## Discussion

Angioleiomyoma, also known as angiomyoma or vascular leiomyoma, is a benign soft tissue tumour comprising mature smooth muscle cells with a prominent vascular component. It was previously classified under ‘smooth muscle tumours’ by the World Health Organization (WHO) in 2002, but reclassified under ‘pericytic tumours’ in the updated classification because of morphological features shared with myopericytoma, that is, showing a perivascular concentric arrangement of smooth muscle cells [3]. Additionally, Morimoto et al. proposed a classification system, that divided angioleiomyoma into solid, cavernous, and venous types and reported that these pathologic subtypes were associated with clinical manifestations [4].

Clinically, angioleiomyoma usually occurs in the lower extremities and appears as a solitary, slow-growing, mobile, firm and occasionally painful cutaneous mass [1]. The pain can often be paroxysmal and provoked by exposure to cold and wind, which is thought to be due to active contraction of smooth muscle that results in local ischaemia [5]. Angioleiomyoma rarely occurs in the lung. Whether patients with angioleiomyoma in the lung have symptoms may depend on the tumour size and location. Symptoms may range from an asymptomatic presentation to recurrent cough when the tumour grows large enough in the lung parenchyma [6, 7].

Preoperative diagnosis of angioleiomyoma is difficult before histopathology [8]. Complete surgical resection is the only way to guarantee curative outcomes [5]. Angioleiomyoma can be correctly diagnosed by microscopy with conventional H&E staining. Special staining for smooth muscle cells, such as actin, desmin, or myosin, and for vascular endothelium, such as factor VIII or CD31 can aid in differentiating angioleiomyoma from haemangioma, angiofibroma, fibroma, angiomyolipoma, and angiomyosarcoma [9]. Attention should also be paid to the possibility of benign metastasizing leiomyoma (BML) when encountering female patients because of BML’s metastatic traits of invading the pulmonary artery [10]. Despite the vascular nature of these tumours, significant bleeding during surgical excision is seldom seen [8]. Recurrence after complete removal is extremely rare, regardless of the pathological subtype [8].

To the best of our knowledge, only two cases of angioleiomyoma of the pulmonary artery have been reported so far [6, 7]. The clinicopathological features of the previously reported cases and our case are summarized in Table 1. Klotz et al. reported a case of angioleiomyoma infiltrated into the left pulmonary artery of a 54-year-old patient who underwent tangential resection of the infringed artery followed by a direct suture of the vessel wall [6]. Terada described a 62-year-old female patient receiving lobectomy for a small mass in the right upper lobe of the lung, in which this mass turned out to be an angioleiomyoma arising from the pulmonary artery [7]. Herein, we report a rare case of angioleiomyoma arising from the RA8b artery in a young male patient who received single-port video-assisted RS8 segmentectomy and we review the literature on angioleiomyoma of the pulmonary artery.

**Table 1:** Clinical and pathological features of angioleiomyomas of the pulmonary artery

	Klotz et al	Terada	Present case
Age/gender	54/female	62/female	27/male
Symptoms	Recurrent cough	None	None
Smoking status	Non-smoker	Non-smoker	Non-smoker
Location	Left upper lobe	Right upper lobe	Right lower lobe
Tumour size (cm)	3.5	1	1.7
Gross view	Tumour mass with the lumen of an artery vessel	Continuous to a pulmonary artery and small pulmonary arteries scattered within the tumour	Tube-like structure with vascular endothelium
Histology	Consisted of spindle cells, arranged in nested bundles	Consisted of mature smooth muscle with acidophilic cytoplasm	Consisted of spindle-shaped smooth muscle cells
Immunostains	Actin, desmin	Actin, vimentin, Ki-67 (2%)	SMA, desmin, Ki-67 (3%)
Treatment	Resection of the whole tumour and pulmonary arterioplasty	Lobectomy	Segmentectomy
Clinical outcomes	Free of disease for 12 months	Free of disease for 10 years	Free of disease for 6 months

## Conclusion

We believe this report emphasizes the necessary awareness of thoracic surgeons that angioleiomyoma should be included in the differential diagnosis of lung neoplasms, Although an accurate preoperative diagnosis can be challenging, in our opinion, CT imaging findings presenting as a soft-tissue density nodule penetrated by the pulmonary blood vessel may indicate the possibility of angioleiomyoma. Due to the benign nature of this tumour, sublobar resection is appropriate for the treatment of angioleiomyoma of the pulmonary artery.


**Additional file 1.** Video 1 Legend. Single-port RS8 segmentectomy was performed through the fifth intercostal space between anterior axillary line and midaxillary line. Insufflation technique was utilized to establish intersegmental border (Asian Cardiovasc Thorac Ann 2019 Nov; 27 [9]). RA8, right anterior basilar artery, RB8, right anterior basilar bronchus, RV8a, lateral branch of right anterior basilar vein.

## Data Availability

As this paper is a case report, all data generated or analysed are included in this article.

## Abbreviations

RA8b: The basal branch of the right anterior basilar artery
RS8: The right anterior basilar segment of the lung
SMA: Smooth muscle actin
WHO: World Health Organization
BML: Benign metastasizing leiomyoma
